# Prediction of Corrosive Fatigue Life of Submarine Pipelines of API 5L X56 Steel Materials

**DOI:** 10.3390/ma12071031

**Published:** 2019-03-28

**Authors:** Xudong Gao, Yongbo Shao, Liyuan Xie, Yamin Wang, Dongping Yang

**Affiliations:** 1 School of Mechatronic Engineering, Southwest Petroleum University, Chengdu 610500, China; gxdgao193@163.com (X.G.); 18380156106@163.com (L.X.); 2 Centre for Infrastructure Engineering, Western Sydney University, Penrith, NSW 2751, Australia; Y.Wang5@westernsydney.edu.au; 3 Technology Inspection Center, China Petroleum & Chemical Corporation, Dongying 257062, China; ydp17@126.com

**Keywords:** API 5L X56 steel material, submarine pipelines, corrosive fatigue crack growth, Paris law constants, stress intensity factor

## Abstract

Corrosive fatigue failure of submarine pipelines is very common because the pipeline is immersed in a sea environment. In Bohai sea, many old pipelines are made of API 5L X56 steel materials, and it is necessary to provide an accurate method for predicting the residual life of these pipelines. As Paris law has been proven to be reliable in predicting the fatigue crack growth in metal materials, the two constants in Paris law for API 5L X56 steel materials are obtained by using a new proposed shape factor based on the analysis of experimental data measured from fatigue tests on compact tension specimens immersed in the water of Bohai sea. The results of the newly proposed shape factor show that, for a given stress intensity factor range (ΔK), the fatigue crack growth rate (*da/dN*) in seawater is 1.6 times of that that in air. With the increase of fatigue crack growth rate, the influence of seawater on corrosive fatigue decreases gradually. Thereafter, a finite element model for analyzing the stress intensity factor of fatigue crack in pipelines is built, and the corrosive fatigue life of a submarine pipeline is then predicted according to the Paris law. To verify the presented method, the fatigue crack growth (FCG) behavior of an API 5L X56 pipeline with an initial crack under cyclic load is tested. Comparison between the prediction and the tested result indicates that the presented method is effective in evaluating the corrosive fatigue life of API 5L X56 pipelines.

## 1. Introduction

Submarine pipeline is regarded as the lifeline of offshore oil and gas transportation. As the offshore oil industry boomed, the frequency of pipeline leaks increased. The submarine pipeline is used in a harsh marine environment for a long time. It is vulnerable to failure due to complex alternating stress and external factors [1,2,3]. The phenomenon of submarine pipeline hanging is inevitable due to the scouring of sea water, and the hanging pipeline is subjected to alternating load under the action of sea wave and it is, hence, sensitive to fatigue failure [4]. Therefore, it is of great significance to study the corrosive fatigue of submarine pipelines in service to prevent the failure. 

The crack growth rate under a given load depends on several factors, including crack length and geometry, temperature, environment, test frequency, and the microstructure of the material. Petit et al. [5,6] have investigated the detrimental effects of atmospheric conditions on fatigue crack propagation in steel, Al and Ti-based alloys. The deleterious effects of water vapor were analyzed based on experiments in a controlled partial pressure atmosphere, and modeling of intrinsic and environmentally assisted crack growth is proposed. At present, many researchers have studied the influence of different environmental conditions on fatigue crack growth rate for different pipelines. Javidi et al. [7] studied the stress corrosion cracking behavior of API 5L X52 steel under the near-neutral and high pH conditions using slow strain rate tests and electrochemical methods. The results revealed that the anodic dissolution at a crack tip was the dominant mechanism at near-neutral pH condition. While at high pH medium, the hydrogen-based mechanism was dominant. Cheng et al. [8] established a two-component model considering anode dissolution and hydrogen embrittlement based on the fracture mechanics theory, and they applied the model for API 5L X65 pipeline steel. The results showed that the model can control the shape of the simulated crack growth curve and the fatigue crack growth (FCG) rate. As the global sustainable development strategy becomes the mainstream, offshore natural gas, which is considered as a clean energy, has attracted the attention of various countries. Therefore, many researchers have studied the FCG rate and the cracking behavior of pipeline steels in different gas environments [9,10,11,12,13,14]. Wang et al. [9] studied the FCG rate of the base metal and the heat affected zone of API 5L X70 pipeline steel under a hydrogen sulphide environment, and they found that the existence of H_2_S greatly accelerated the FCG rate. Zheng et al. [10] also obtained a similar law in the study of API 5L X56 pipeline steel under an H_2_S environment. In addition, it is found that the presence of gaseous hydrogen also has a great impact on the FCG rate of steel, which exceeded an order of magnitude larger than that of the FCG rate under laboratory air environment [11,12,13,14]. Amaro et al. [11] studied API 5L X100 pipeline steel in high-pressure gaseous hydrogen environments, and they found that the FCG response in hydrogen at *da/dN* < 3 × 10^−4^ mm/cycle is primarily affected by the hydrogen concentration within the fatigue process zone, resulting in a hydrogen-dominated mechanism, and the FCG response in hydrogen at *da/dN* < 3 × 10^−4^ mm/cycle results from fatigue-dominated mechanisms. 

API 5L X56 pipeline steel is now used in the submarine pipelines in the Bohai sea of China. Corresponding studies on X56 submarine pipeline steel have also been carried out in the literature [10,15,16]. Yi et al. [15] simulated stress corrosion cracking and hydrogen permeation behavior of X56 pipeline steel under atmospheric environment containing SO_2_, and the results show that hydrogen ion concentration decreases with time, indicating that the hydrogen concentration on the surface decreases with the formation of corrosion rust. As the passivation film dissolves, the hydrogen permeation rate increases and the stress corrosion cracking sensitivity of API 5L X56 increases with the increase of SO_2_ concentration. Zhu et al. [16] studied the permeation action of API 5L X56 steel in the sea mud with and without sulfate-reducing bacteria at corrosion and cathodic potential were studied with an improved Devanathan–Stachurski electrolytic cell. The above studies on the API 5L X56 submarine pipeline are all influenced by a single factor, and the fatigue remaining life of the pipeline with initial cracks in service is not analyzed. 

Structural cracking is a major problem for large infrastructures including dams, bridges, submarine pipelines and offshore wind turbine foundations. At the same time, reliability analysis has become a concern of researchers. Based on the probability theory, reliability analysis of fatigue fracture for engineering structures and components has been conducted, and proposed several analysis models. Commonly used techniques include Monte Carlo simulation method and first-order reliability method [17]. In Monte Carlo simulation, the random variable is formed by its distribution function. For each Monte Carlo simulation sample, the sensitivity of each stress intensity factor (SIF) to the random variable is calculated and compared with the obtained SIF to evaluate whether failure will occur. Based on the above method, the fatigue life of the pipeline is analyzed [18,19,20]. An *S-N* curve based on damage mechanics is another way to predict fatigue life. The *S-N* curve is used to predict the fatigue life of crack in suspended pipeline. Wormsen et al. [21] carried out tests on low alloy machined steel forgings and analyzed *S-N* curves in air and seawater environments with cathodic protection measures. They provided specific plans for fatigue tests of submarine pipelines. Cunha et al. [22] introduced an *S-N* curve from tensile test material properties, and they proposed a new algorithm to evaluate the fatigue life of dented pipelines. Hong et al. [23] collected ASTM standard specimens from the API 5L X42 gas pipeline, and FCG tests were subsequently performed. Additionally, *S-N* curve of each specimen was estimated to verify the accuracy of the prediction model. Dong et al. [24] analyzed the weld data recorded in a large number of literatures from 1947 to present by means of the new mesh-insensitive structural stress method and the associated master *S-N* curve approach. According to the characteristics of fracture mechanic materials, Lukács et al. [25,26] evaluated and calculated the reliability of different pipeline structural unit models, during which different crack geometries were assumed. Meanwhile, the stress intensity factors and safety factors were also determined for all cases. Both methods are based on empirical formulas, model analysis and finite element simulation to obtain the fatigue life. It is difficult to verify the accuracy of the predicted results in practical application.

The methods for predicting fatigue life of metal materials or structures can be divided into *S-N* curves based on damage mechanics and Paris law based on fracture mechanics. At present, Paris law-based on fracture mechanics is used to predict the corrosion fatigue life of cracked parts. The main reason is that *S-N* curve based on damage mechanics is obtained by applying alternating loads on smooth and non-cracked specimens. In fact, defects are inevitable when metal is processed, and so the predicted life from S-N curve is dangerous. Paris law based on linear elastic fracture mechanics analyzes the remaining life of cracked components, which is more instructive for practical engineering. Moreover, Paris law can reflect the relationship between fatigue crack growth rate (*da/dN*) and stress intensity factor range (ΔK). It is only necessary to measure the Paris law constants (*C* and *m*) in a certain environment through FCG tests. In this study, finite element analysis is carried out to analyze the stress intensity factors under different crack depths in API 5L X56 submarine pipelines. The fatigue crack growth life is then predicted from Paris law. To verify the accuracy of the prediction method, a full-scale size API 5L X56 pipeline was tested in a fatigue loading condition. The effect of seawater on the crack growth rate of API 5L X56 steel was also studied under a laboratory environment, and a new method for predicting the fatigue life of API 5L X56 pipeline life was proposed.

## 2. Test on Fatigue Crack Propagation of API 5L X56 Steel Material

### 2.1. Mechanical Properties of API 5L X56 Steel Material

Fatigue crack growth test is based on Paris law to establish the relationship between the FCG rate (da/dN) and the stress intensity factor range (ΔK). For a linear elastic steel specimen, the SIF is linearly related to the load and depends on the geometry and size of the steel specimen and the crack. Coupons test and Charpy pendulum impact test are both carried out to obtain the mechanical properties of API 5L X56 steel materials including the elastic modulus (*E*), the yield strength (*σ*_y_), Poisson’s ratio (*ν*), and the fracture toughness ($K_{IC}$). The impact energy is measured according to GB/T229-2007 standard for “Metallic materials—Charpy pendulum impact test method” [27], and the experimental results are listed in Table 1.

The fracture toughness of API 5L X56 steel material is estimated according to Equation (1) provided in BS7910-2013 standard for “Guide to methods for assessing the acceptability of flaws in metallic structures” [28] as follow

(1) $$K_{\mathrm{IC}}=[(12\sqrt{C_{V}}{-20)(25/B)}^{0.25}]+20,$$

where $C_{V}$ is the Charpy impact energy, B is the thickness of the specimens. Based on the data in Table 1, the fracture toughness of API 5L X56 steel material can be calculated. Combined with the tensile test of metal materials, the basic mechanical properties of API 5L X56 steel material are listed in Table 2.

### 2.2. Design of C(T) Specimens

**Figure 1:**
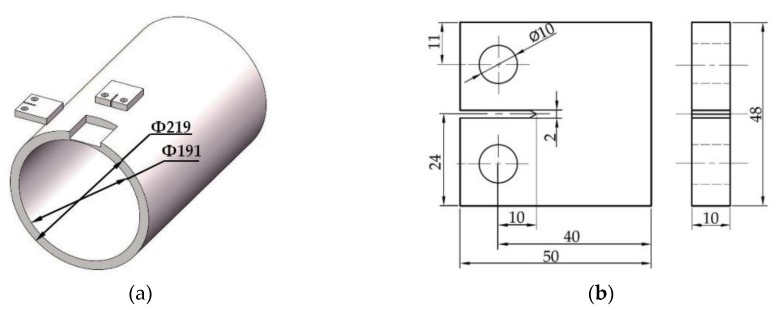
Extraction location and dimensional drawing of C(T) specimens: (**a**) Crack plane orientation; (**b**) C(T) specimen.

The fatigue crack growth test is the most commonly used method to study the FCG rates of various homogeneous metal materials. The material used in the C(T) specimens is API 5L X56 steel material which is obtained from the submarine pipelines in Bohai sea. Standard compact tension, C(T), specimens are extracted from submarine pipeline with a diameter of 219 mm and a wall thickness of 14 mm. Figure 1a shows that the plane direction of the crack is perpendicular to the axial direction and parallel to the axial direction respectively. Li et al. [29] carried out a load-controlled three-point bending test on API 5L X80 steel material. The results show that the FCG rate of the specimens from different directions are basically the same. In this study, the sampling direction of C(T) specimens is no longer distinguished.

C(T) specimens are designed according to GB/T 6398-2000 standard for “Metallic materials―Fatigue testing―Fatigue crack growth method” [30] and ASTM E647-13 [31]. The wall thickness of API 5L X56 pipeline is 14mm. Under the premise of guaranteeing the machining allowance, the thickness of C(T) specimens is selected as *B* = 10 mm and the width is selected as *W* = 40 mm. Figure 1b is a standard C(T) specimen used in fatigue crack growth test. Overall, three specimens are tested on the STM793 fatigue testing machine, and the specimen marked with X56-A-1 represents the one tested in an air environment while the other two specimens (marked with X56-S-1 and X56-S-2) are tested in Bohai sea environment.

The FCG tests are carried out on the STM793 fatigue testing machine, which is composed of a loading device and control system. The loading device is used to provide cyclic load, while the control system is used for controlling loading process and collecting testing data. All specimens are fabricated to produce a sharp crack with a certain length of approximately 3 mm from the machined V-notch using the K-decreasing technique to ensure that the K equation is not affected by the initial notch shape. The stress-intensity factor range is stepped down at a constant rate as the precracked length increases, and the final ΔK equals to the minimum ΔK at the end of the precracking. Precracking in air at a frequency of 10 Hz at room temperature, and the crack length at the end is recorded as the initial crack length, $a_{0}$. Thereafter, the constant amplitude loading stage for fatigue crack growth test is followed. The test is terminated when the FCG rate exceeded 0.01 mm/cycle, and the crack length at this time is called the final crack length, $a_{f}$.

### 2.3. Parameter Selection in Fatigue Test

The tests are carried out in both air and seawater conditions, and the seawater used in the test is taken from Bohai sea of China where the submarine pipeline is located. C(T) specimens tested in a seawater environment are soaked in seawater for more than 24 hours before precracking. All FCG tests in air and in seawater environments are loaded in a sinusoidal wave form with a stress ratio of 0.1 and a constant amplitude. It is noted here that, even though the applied load ratio experienced by the submarine pipeline may be negative, a previous study in the literatures [32,33,34] has found that the FCG rates at *R*-ratio of −1.0 is similar or lower than that at *R* = 0.1 for steel in air and in seawater environments. Therefore, the *R*-ratio of 0.1 used in this study to characterize the FCG rate is relatively conservative. Previous study indicated that the effect of loading frequency, f, on the fatigue crack growth tests varies from environment to environment [35]. To simulate the actual seawater environment as much as possible and to consider the efficiency problem, the loading frequency in this seawater test environment is 0.5 Hz. Previous studies found that the influence of loading frequency on the FCG rate of steel in the air environment is negligible [34,36,37]. Therefore, FCG test in the air is performed at a frequency of 10 Hz at room temperature. When selecting the maximum load, $P_{\max}$, the load should not be too large to guarantee that the crack tip is in the small yielding range to meet the requirements of Linear and Elastic Fracture Mechanics (LEFM). Simultaneously, the load should not be too small to ensure that ΔK is greater than the stress intensity factor threshold, $△K_{\mathrm{th}}$. In addition, the selection of the load is also within the effective control range of the fatigue testing machine. Testing parameters such as loading frequency, f, maximum loading load, $P_{\max}$ and load ratio, *R*, are shown in Table 3.

### 2.4. Crack Growth Monitoring

**Figure 2:**
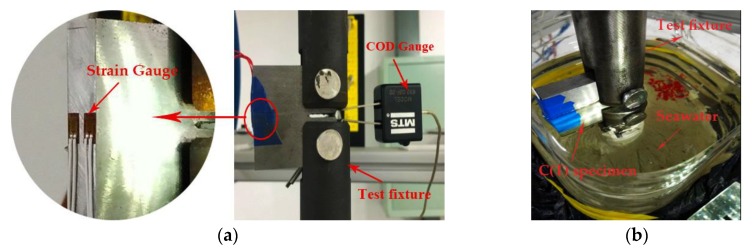
Fatigue crack propagation monitoring method: (**a**) Monitoring of crack growth in air environment; (**b**) Monitoring of crack growth in seawater environment.

In the FCG tests, a flexible method is used to measure the crack length development. Different monitoring methods are used in different environments. In the air environment, the crack propagation is monitored by using the Crack Opening Displacement (COD) gauge and the Back Face Strain (BFS) method. Figure 2a shows the position of the COD gauge and strain gauge. Previous study in the literature [35] verified the accuracy of the COD and BFS method for monitoring crack growth, and the results showed that the BFS method can be used to replace the COD method. In this study, the test data of API 5L X56 pipeline steel collected from two monitoring methods are compared to verify the reliability of those two methods. Due to the difficulty in installing COD gauges in a seawater environment, only the BFS method is used to monitor FCG in this condition. For the fatigue test in seawater, the strain gauge surfaces are coated with a layer of AZ-710 anti-corrosive glue to protect the strain gauge from the effect of corrosion. After the glue is solidified, a layer of waterproof tape is applied to achieve double protection, as shown in Figure 2b. In addition, it should be noted that the load frequency is up to 10 Hz. Therefore, the TST5912 dynamic signal test and analysis system is used for data acquisition. During the cyclic loading process, the acquisition frequency of the dynamic strain gauge in seawater is set to 100 Hz. 

### 2.5. Fatigue Crack Propagation

Paris law is commonly used to describe the fatigue crack propagation relationship, i.e., the relationship between the FCG rate (*da/dN*) and the stress intensity factor range (ΔK). The stress intensity factor range can be defined as follows:

(2) $$\Delta {K\;=\;K}_{\max}\;{-\;K}_{\min},$$

where *K* is the stress intensity factor, which is a parameter in linear and elastic fracture mechanics and it is defined as follows:

(3) $$K\;=\;F\sigma \sqrt{\pi a},$$

where σ is the nominal stress (the applied stress), a is the crack length, and *F* is a dimensionless shape function. It is indicated clearly in the GB/T 6398-2000 standard that the SIF range of C(T) specimens is calculated from the following equation:

(4) $$\Delta K\;=\;\frac{\Delta P}{{BW}^{1/2}}g(\frac{a}{W}),$$

where *P* is the applied load at both ends of the C(T) specimen, *B* is the specimen’s thickness, and *W* is the specimen’s width. The solution of the shape function for a standard C(T) specimen is given in References [30,31] as follows:

(5) $$g(\frac{a}{W})\;=\;\frac{(2\;+\;\alpha )(0.886\;+\;4.64\alpha \;-13.32\alpha^{2}\;+\;14.72\alpha^{3}\;-\;5.6\alpha^{4})}{{\left(1-\alpha \right)}^{3/2}},$$

where α=a/W. Equations (2) and (4) derive the equation of the SIF range as follows:

(6) $$\Delta {K\;=\;K}_{\max}{-K}_{\min\;}{=\;(P}_{\max}{-P}_{\min})\frac{1}{{BW}^{1/2}}g(\frac{a}{W}),$$

where d*a*/d*N* is plotted against ΔK in log–log axes, and general three regions can be observed in FCG trend. The three regions are named as the fatigue crack initiation (region I), the stable crack growth stage (region II), and the unstable fracture stage (region III). In region I, the SIF is lower than the stress intensity factor threshold, $\Delta K_{\mathrm{th}}$, and there is no crack growth. When ΔK exceeds stress intensity factor threshold, it is in region II and the crack begins to grow. It is worth pointing out that the crack growth follows a power law, namely the so-called Paris law, as given in Equation (7) in region II. When ΔK increases to a certain value, it enters region III, where $K_{\mathrm{Imax}}$ is close to the $K_{\mathrm{Ic}}$ of the material, and the FCG rate increases sharply until the fracture.

(7) $$\frac{da}{dN}{\;=\;C(\Delta K)}^{m},$$

where *C* and *m* are material constants related to the test conditions, and they can be obtained by regression analysis of FCG test data.

Two methods for calculating the FCG rate (*da/dN*) are recommended in GB/T 6398-2000. One is the secant method and the other is the seven-point incremental polynomial method. Considering that the secant method is relatively simple to operate, it is used to calculate the FCG rate in this study.

## 3. Analysis of Fatigue Crack Growth

According to the test conditions given in Table 3, fatigue tests are carried out in air and seawater corrosive environments to obtain the crack propagation process. It is worth noting that the dynamic strain gauge output is the relationship between time and strain when using the BFS measurement method in the fatigue tests. Therefore, it is necessary to obtain the relationship between the strains and the corresponding crack lengths of C(T) specimen. Such relationship is obtained through finite element analysis by using ABAQUS software, and the calculation results are listed in Table 4. The convergence analysis is carried out to analyze a C(T) specimen with a crack length of 13 mm, and it is found that the result converges when the element size is 0.7 mm. Based on the numerical results of the crack length and the back strain listed in Table 4, a fifth-order polynomial, which is presented through a curve fitting technique by using DataFit software, is used to derive the relationship between the crack length and the strain, and the detailed expression of the polynomial is listed as follows [34]:

(8) $${a\;=\;C}_{0}{\;+\;C}_{1}{(\mu \varepsilon \;)+\;C}_{2}{\left(\mu \varepsilon \right)}^{2}{+\;C}_{3}{\left(\mu \varepsilon \right)}^{3}{+\;C}_{4}{\left(\mu \varepsilon \right)}^{4}{+\;C}_{5}{\left(\mu \varepsilon \right)}^{5},$$

where $C_{0}{\;\mathrm{to}\;C}_{5}$ are the regression coefficients. The curve fitting results are shown in Table 5, and its fitting accuracy can be assessed from the value of R^2^ (much close to 1.0).

In addition, Adedipe et al. [35] established a relation between crack length and strain considering such factors as specimen’s material (elastic modulus *E*), specimen’s thickness *B*, specimen’s width *W*, and the applied load *P*, which mathematical expression is represented by the polynomial as follows:

(9) $${a/W=C}_{0}{\;+\;C}_{1}{U\;+\;C}_{2}U^{2}{\;+\;C}_{3}U^{3}{\;+\;C}_{4}U^{4}{\;+\;C}_{5}U^{5},$$

where a/W is the aspect ratio of the C(T) specimen and $C_{0}{\;\mathrm{to}\;C}_{5}$ are the regression coefficients related to the crack length to width ratio.

(10) $$U=[(\left|EB\varepsilon W\right|{/P)}^{1/2}{+1]}^{-1}$$

where |EBεW|/P is a back surface strain parameter. Compare the data calculated from Equations (8) and (9); the method with higher precision to determine the crack length can be selected.

**Figure 3:**
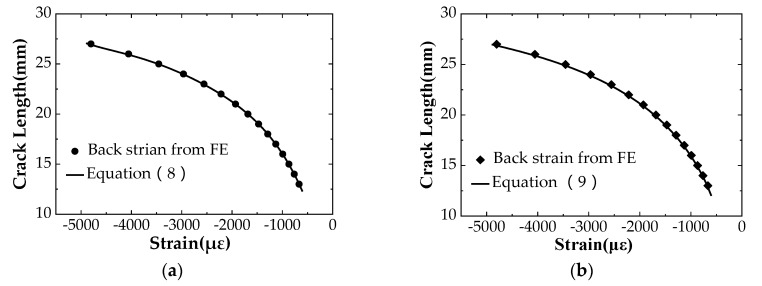
Relation between crack length and strain value measured by the BFS (Back Face Strain) compliance method: (**a**) Fitting results from Equation (8); (**b**) Fitting results from Equation (9).

**Figure 4:**
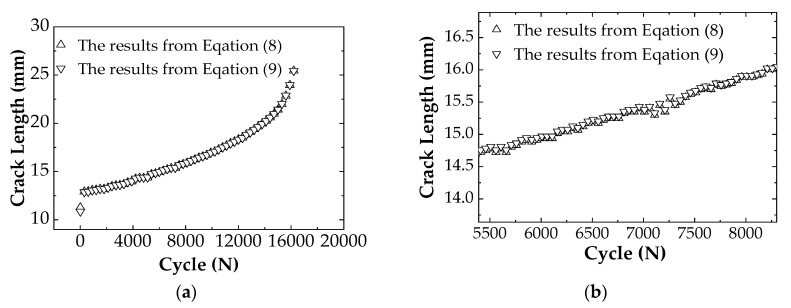
Relation between crack length and strain value measured by the BFS compliance method: (**a**) Comparison of the results from Equations (8) and (9); (**b**) Enlarged view of Figure 4a.

Figure 3 shows the fitting results of the crack length and the strain value of the C(T) specimen using Equations (8) and (9), respectively. The crack lengths calculated from Equation (8) and from Equation (9), respectively, are plotted in the same coordinate system for comparison, as shown in Figure 4. It can be seen that the crack lengths calculated by the two formulas are almost the same. From the data analysis, the results calculated from Equation (8) are relatively bigger, i.e., the predicted life is safe. Therefore, Equation (8) is used to predict the crack propagation in this study.

**Figure 5:**
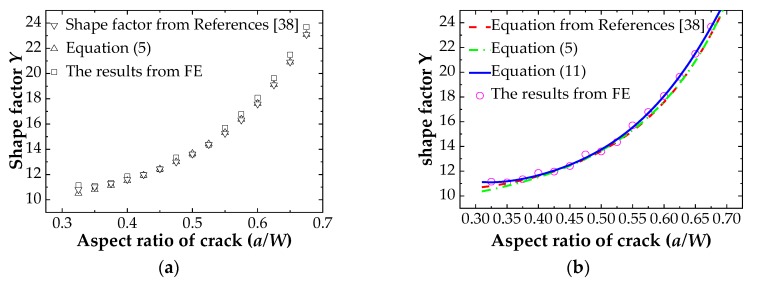
Shape factor error analysis: (**a**) Numerical simulation compared with recommended shape factor in the standard; (**b**) Comparison of new shape factor equation and old equation.

The purpose of FCG tests is to obtain the material constants C and m of the API 5L X56 pipeline from Paris law and, thus, the relationship between the stress intensity factor range ΔK and the FCG rate *da/dN* can be determined. The GB/T 6398-2000 [30] and ASTM E647-2013 [31] standards indicate that the shape function can be calculated using Equation (5) for a standard C(T) specimen with a/W ≥ 0.2. However, previous studies in the literature [38,39] pointed out that the shape function solution of the standard C(T) specimen does not follow the equation provided by standards [30,31] for shallow cracks with a/W ≤ 0.4. To reduce the error caused by the shape factor, the shape factor of the standard C(T) specimen is evaluated by numerical simulation before test data processing. The SIFs of cracks with different lengths in the standard C(T) specimens are calculated numerically by using the contour integral technique. The numerical values of the shape function are compared with the results from Equation (5) and the new shape factor proposed in [38] as shown in Figure 5. A shape function calculation equation suitable for this test is proposed.

Figure 5a analyzes the shape factor of the standard C(T) specimen made of API 5L X56 submarine pipeline material. The results show that the ABAQUS simulation is higher than the results calculated from Equation (5) and from the new equation proposed in Reference [38]. It can be found clearly that the shallow crack with a/W ≤ 0.4 has a larger error. Hence, the equation recommended in the standard cannot be used in this study. The data obtained from numerical simulation is fitted by using a polynomial, and a new shape factor equation suitable for calculating the stress intensity factor range, ΔK, of the material is presented as expressed in Equation (11). The fitting degree of R^2^ is 0.9983. Figure 5b shows the comparison of the results calculated from different equations.

(11) $$Y\;=\;-1562.078\alpha^{5}+\;4225.567\alpha^{4}-\;4294.602\alpha^{3}+\;2164.728\alpha^{2}-\;538.762\alpha +\;63.495,$$

## 3.1. FCG Behavior of API 5L X56 Material in Air

**Figure 6:**
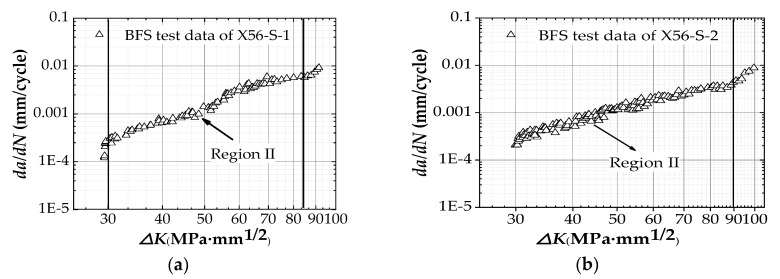
Tested data points in log–log axes (contains the data of region I): (**a**) Specimen X56-S-1; (**b**) Specimen X56-S-2.

The fatigue crack growth tested in the air is monitored by the COD gauge method and the BFS method, both of which belong to the flexible method. To verify the accuracy of the two measurement methods, the collected data of specimen X56-A-1 are analyzed and compared. The data collected from the COD gauge is processed by the STM793 control system, and the relationship between the FCG rate, *da/dN*, and the SIF range, ΔK, is finally displayed on the log–log axes. The data collected from BFS method needs to be transferred. The threshold stress intensity factor range value ($\Delta K_{th}$) is used for the assessment of the used initial stress intensity factor range values [40,41]. After processing the data (contains the data of region I), it can be seen in Figure 6 that the FCG trends observed in X56-S-1 and X56-S-2 specimens suggest that the threshold stress intensity factor range for X56 steel in seawater is approximately $\Delta K_{th}$ = 27 $\mathrm{MPa}\cdot \sqrt{m}$. In addition, the threshold stress intensity factor range of X56 steel in the air environment was directly inquired from the fatigue testing machine, $\Delta K_{th}$ = 28 $\mathrm{MPa}\cdot \sqrt{m}$.

**Figure 7:**
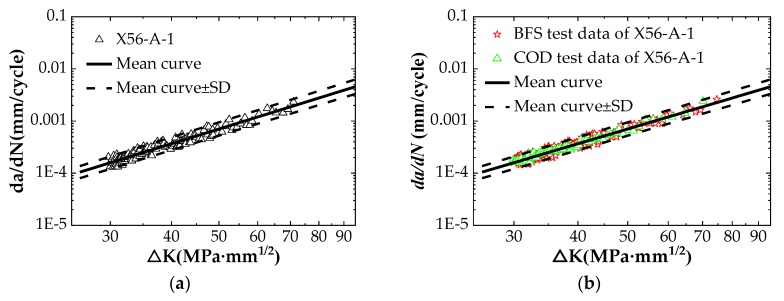
Comparison of different crack growth monitoring methods: (**a**) FCG test data obtained by BFS method; (**b**) Comparison of test results of COD and BFS.

When processing BFS data, the data points that mainly exhibit linear behavior are selected, and they are used to draw the average curve and the upper and the lower limits on the log–log axes (average curve ± SD). All the test points are re-selected by using FORTRAN compiler software, and the data outside the upper/lower limit are eliminated. The relationship between the FCG rate, *da/dN*, and the stress intensity factor range, ΔK, is shown in Figure 7a. The relationship between the FCG rate and the stress intensity factor range obtained by the two flexible methods is shown in Figure 7b. It shows that the data monitored by the COD method and by the BFS method have a high degree of coincidence, indicating that both methods can achieve the purpose of monitoring crack length. This result provides a basis for monitoring crack length using the BFS method alone in seawater experimental tests. The data are measured in two ways to calculate the material constants *C* and *m* of Paris law, and the calculated results are summarized in Table 6.

## 3.2. FCG Behavior of API 5L X56 Material in Seawater

**Figure 8:**
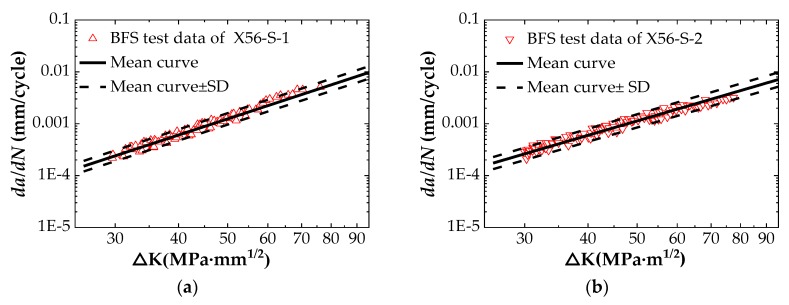
Tested data points in log–log axes: (**a**) Specimen X56-S-1; (**b**) Specimen X56-S-2.

Since the presence of seawater increases the difficulty of the fatigue test, the crack length is monitored in the seawater environment by using only BFS method. Table 3 shows that fatigue tests on two specimens are conducted in seawater to verify the accuracy of the tested results. The two sets of data are preprocessed separately, and the processing method is the same as the BFS method data processing method in air. The relationship between the FCG rate, *da/dN*, and the stress intensity factor range, ΔK, is shown in Figure 8. Figure 8a,b show the trend between the FCG rate and the stress intensity factor range for specimens X56-S-1 and X56-S-2, respectively. As seen in Figure 8, the relationship between *da/dN* and ΔK exhibits a linear change in log–log axes. According to this relationship, the two sets of tested data are fitted linearly to obtain the Paris law constants (C and m) of the API 5L X56 pipeline steel under free corrosion conditions, as shown in Table 6. The Paris law constants obtained from the two sets of data are used for fatigue life prediction of the standard C(T) specimen, and then the prediction results are compared with the tested results in Table 6 to judge the accuracy of the prediction method. The results show that both sets of data can be used to predict fatigue life of a standard C(T) specimen. Since the predicted results of specimen X56-S-2 are closer to the experimental value, the Paris law constants measured by X56-S-2 specimen are used to predict the corrosion fatigue life of the submarine pipeline. 

## 3.3. Comparison of FCG Behavior in Air and in Seawater

**Figure 9:**
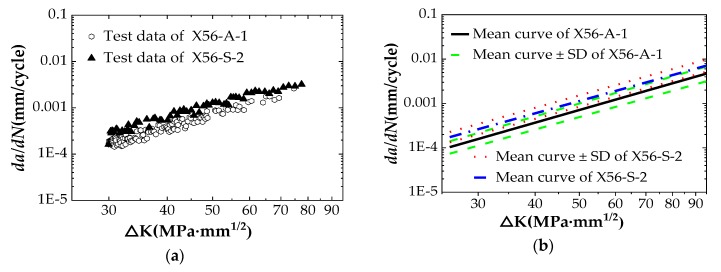
Comparison of FCG rates of API 5L X56 steel in seawater and in air: (**a**) Comparison of tested data on log–log axes; (**b**) Comparison of fitting curves on log–log axes.

The FCG data tested in air and in seawater are plotted together in the same log–log axes as shown in Figure 9. Figure 9a shows a comparison of the tested data, and Figure 9b shows a comparison of fitting curves (with standard deviation). It indicates that the da/dN−ΔK curve in air is consistent with the trend of steady fatigue crack growth in region II, i.e., the FCG rates increases linearly when the stress intensity factor range increases. After calculation, the FCG rate, *da/dN*, corresponding to the stress intensity factor range ΔK = 29.64 $\mathrm{MPa}\cdot \sqrt{m}$ (the initial crack length of the X56-A-1 specimen is 12.98 mm) is 1.53 × 10^−4^ mm/cycle. At the end of the test, the FCG rate corresponding to the stress intensity factor range ΔK = 75.06 $\mathrm{MPa}\cdot \sqrt{m}$ (crack termination length $a_{f}$ = 25.03 mm) is 2.4 × 10^−3^ mm/cycle.

For API 5L X56 pipeline material, there are similar laws in seawater as in air. The difference from the air environment is that the FCC rate of the specimen in the seawater is increased faster, and the slope of the da/dN−ΔK logarithmic curve in the seawater is small. This indicates that the influence of seawater corrosion on the FCG rate is greater in the initial stage of fatigue crack growth. With the increase of ΔK, the influence of seawater corrosion is reduced gradually, and the FCG rate in the seawater is closer to the FCG rate in the air. 

According to Table 6, *da/dN* = 2.57 × 10^−4^ mm/cycle corresponding to $a_{0}$ = 12.98 mm in seawater is obtained, which is about 1.68-times the FCG rate in air. The corresponding *da/dN* = 3.63 × 10^−3^ mm/cycle at $a_{f}$ = 25.03 mm is about 1.52-times the FCG rate in air. From the whole test process, the FCG rate of API 5L X56 pipeline material in seawater is about 1.6-times that in air. However, the influence of seawater on corrosion fatigue is reduced gradually with the increase of FCG rate.

## 4. Prediction of Corrosion Fatigue Life of Submarine Pipeline with Initial Crack Defect

Based on Paris law and the finite element analysis for SIF, an approximate integration method is presented for predicting the fatigue life of a submarine pipeline with an initial crack defect. To verify the effectiveness of the presented method, a corresponding experimental test on a full-scale API 5L X56 pipeline is carried out in laboratory.

### 4.1. Experimental Test on Corrosion Crack Growth of a Full-Scale Submarine Pipeline

**Figure 10:**
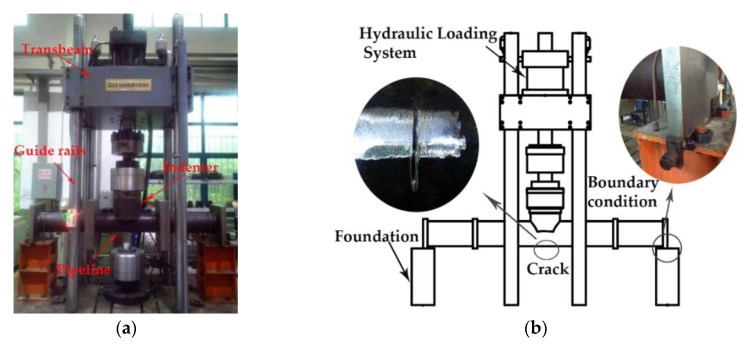
Fatigue test on a full-scale submarine pipeline: (**a**) Fatigue test equipment for full-scale pipeline; (**b**) Schematic view and details of fatigue crack test.

The corrosion fatigue crack propagation test on a full-scale API 5L X56 submarine pipeline is carried out to provide an actual basis for the prediction of corrosion fatigue life. The API 5L X56 submarine pipeline used in the test is fabricated in accordance with API SPEC 5L [42] issued by the American National Standards Institute. The structure of the fatigue testing machine and the clamping of the specimen are shown in Figure 10. In addition, the prefabricated surface crack shown in the figure is located on the bottom surface of the specimen at the mid-span, and it is prefabricated by a circular abrasive cutting wheel to produce a surface crack with a width of 1.5 mm, a length of 52.1 mm and a depth of 5.5 mm. The pipeline is pinned at both ends on the supports, and a pressure in the sinusoidal wave is applied at the mid-span. To ensure that the crack can expand during the fatigue loading process, the amplitude of the cyclic loading must make the crack tip has a certain stress intensity factor to drive the crack propagation and ensure that the pipeline does not produce a wide range of yield. The amplitude of the cyclic load in the fatigue test is 235 kN. The loading frequency, waveform, stress ratio, and other parameter settings are consistent with the FCG tests of the API 5L X56 C(T) specimens. The specific experimental parameters are shown in Table 3. 

**Figure 11:**
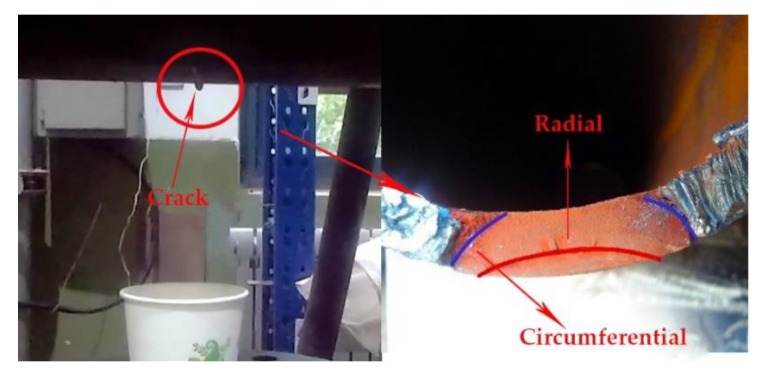
Surface of fatigue crack.

To simulate the seawater conditions, natural seawater is continuously injected into the crack-containing part during the test. At the same time, non-corrosive clean water is injected into the pipeline to monitor the penetration of the fatigue crack. A large amount of water leakage occurs when the fatigue crack is through the tube wall. At the left of Figure 11, the crack has been penetrated and the water starts to drip. After the end of the fatigue test, the crack surface is visible in the cut pipe as shown in Figure 11. It can be seen from the fatigue crack surface that the fatigue crack expands in both length and depth direction. However, the expansion rate does not change uniformly during the fatigue crack propagation. In the beginning, the initial crack propagates faster along the depth direction of the pipeline, while the crack propagates relatively slowly along the length. As the crack depth increases, the FCG rate along the circumferential direction increases.

### 4.2. Prediction on Fatigue Life by Using Paris law

When the safety evaluation of crack-containing components is carried out, the material constants of Paris law are only related to the test environment and materials, and they are widely used for the prediction of fatigue life. The life prediction can be performed by integrating Paris law as shown in Equation (12).

(12) $$\int_{a_{i}}^{a_{i+1}}\frac{da}{{C(\Delta K)}^{m}}=\int_{N_{i}}^{N_{i+1}}dN,$$

where $a_{i}$ and $a_{i+1}$ are the crack depths of any two cracks in the stable crack growth region, $N_{i}$ and $N_{i+1}$ are the number of cycles corresponding to the crack length is $a_{i}$ and $a_{i+1}$.

Equation (12) shows that the fatigue life of the crack in region II can be predicted if the initial crack length ($a_{0}$) and the final crack length ($a_{1}$) are known. The ΔK of the C(T) specimen can be calculated according to Equation (5). In this paper, the new shape factor, which is calculated from the presented equation based on finite element analysis, is used to calculate ΔK. The accuracy of the Paris law constants obtained in the FCG test directly affects the prediction of the corrosion fatigue life of the submarine pipeline. ΔK is calculated using two shape factor equations, and the corresponding Paris law constants are fitted based on this. Using Equation (12) to obtain the number of cycles of the C(T) specimen and comparing it with the test results, it was found that the error was within 10% as listed in Table 6. Using Equations (5) and (11), respectively, to calculate the Paris law constants, it is found that the power exponent constants calculated from Equation (5) are usually smaller than the calculated results from the new equation of shape factor. Table 6 shows that predicting the fatigue life of the C(T) specimen using Equation (5) recommended in the standard produces more conservative predictions. In contrast, the results calculated from Equation (11) can be used for expectations of the remaining life of the specimens with a better result.

### 4.3. Finite Element Analysis for ΔK of Fatigue Crack in Submarine Pipeline

In recent years, finite element analysis using commercial software has been favored by researchers because of its simple operation, low cost and strong applicability. ABAQUS is used here to carry out numerical analysis for the stress intensity factor range (ΔK) of the C(T) specimen, and the numerical results are compared with the experimental values. It is found that the finite element analysis can obtain better results. Therefore, ABAQUS is also used to carry out finite element analysis to analyze the cracked pipelines. When using finite element software to simulate the crack propagation process of submarine pipelines, the crack propagation process in the pipeline can be divided into several stages, and the crack propagation life at the end of a certain stage can be used to predict the crack propagation life at this stage. This method is called approximate integration method. In addition, there are growth behavior in both the radial direction and the circumferential direction during the fatigue crack growth process of the pipeline, and the FCG rates are different. 

**Figure 12:**
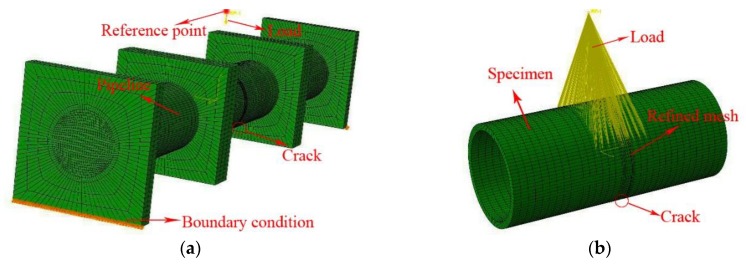
Finite element model of pipeline with initial crack: (**a**) Schematic view of FE model; (**b**) Details of the FE model.

The established finite element model for cracked pipeline needs to calculate the SIF values along the crack front. Such SIFs can be used in Pairs law to calculate the crack growth rate and rate ratio in different directions. According to the ratio of the circumferential direction to the radial direction, the circumferential fatigue crack direction of the pipeline can be matched. Depending on the ratio of the radial and the circumferential FCG rates, the length of the cracks along the radial and the circumferential directions can be matched. Because of the stress singularity at the crack tip, a 15-node iso-parametric stress singular element is required for simulating the crack tip using the perimeter integral technique. Except for the crack front region, the other regions are meshed with a 20-node hexahedron. The reduced integration technique is introduced in the calculation process to improve the calculation efficiency. The loading and the boundary conditions in the finite element analysis are as consistent as possible with the testing conditions, and the finite element model of the pipeline are shown in Figure 12.

During the propagation process of the corrosion fatigue crack, the crack propagates along the radial and the circumferential directions of the pipeline and the expansion rate is different. Taking the X56-S-2 specimen as an example, the cracked pipeline needs to establish a finite element model with different crack lengths in two directions for analysis. The stress intensity factor range corresponding to different crack lengths are shown in Table 7 and Table 8, respectively.

Table 6 shows that the submarine pipeline experienced a total of 42079 loading cycles during the corrosion fatigue test. The final crack parameters corresponding to the number of loading cycles are as follows: the crack propagation in radial direction (crack depth) is 13.9 mm, and the crack propagation in circumferential direction (crack length) is 60.2 mm. The final crack parameters simulated from finite element analysis are as follows: the crack propagation in radial direction is 13.2 mm, and the crack propagation in circumferential direction is 59.5 mm. The numerical simulation produces an accurate enough prediction on the fatigue crack propagation, which indicates that the presented finite element model is suitable for analyzing the SIFs of the crack in a pipeline.

### 4.4. Analysis of Corrosion Fatigue Crack Propagation

**Figure 13:**
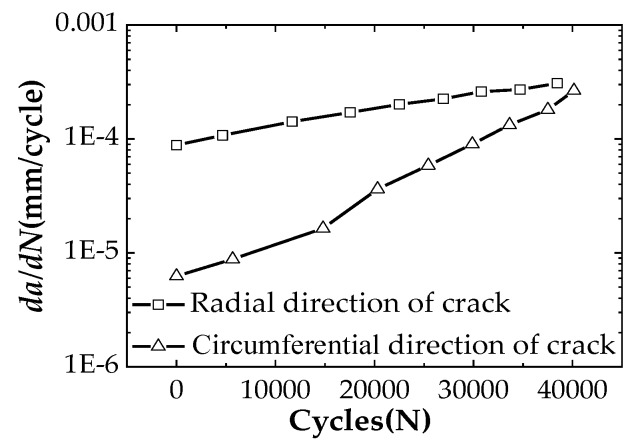
FCG rates in the radial and circumferential direction of the pipeline.

When fatigue crack propagates in the pipeline, it is found that the crack growth rates in the radial direction and in the circumferential direction are not the same. To reflect the expansion rates of the crack in the depth direction and in the length direction more clearly and intuitively, the data in two different directions are compared in Figure 13. The figure shows that the initial crack grows faster along the radial direction of the pipeline at the beginning of the FCG test while the crack propagates relatively slowly along the circumferential direction. As the crack depth increases to a certain extent, the growth of the crack along the circumferential has a certain hindrance to the radial expansion, and the rate of crack propagation along the circumferential increases, i.e., the slope of the curve becomes larger when the number of cycles in the figure is approximately 15,000. Subsequently, when the crack grows to a certain extent in the circumferential direction, the FCG rate in that direction will decrease. The number of cycles per stage is accumulated to calculate the fatigue life of the pipeline extending in different directions. Numerical analysis of radial crack propagation revealed that the crack experiences 40,935 loading cycles when it propagates from 5.5 mm to 13mm in depth, which results in an error of −2.7% compared to the experimental measurement. The crack experiences a total of 39,383 loading cycles in circumferential direction from 52 mm to 58 mm based on numerical simulation, which has an error of −6.4% compared to the experiment measurement. If the life prediction is carried out from Equation (5), the above two errors are −8.6% and −12.9%, respectively.

The predictions show that the predicted values from two equations are smaller than the experimental values, indicating that the predicted results are within the safe life of the cracked pipeline, and the predictions have guiding significance for the corrosion fatigue life evaluation for pipelines. In addition, it is found that the results predicted by the new equation with the shape factor presented in this study are closer to the experimental values while the predictions based on the shape factor recommended in the standard are too conservative. In summary, through the combination of FCG test and the finite element simulation, fatigue crack growth in a pipeline can be predicted accurately and effectively. It is also worth noting that the fatigue life prediction needs to verify the shape factor equation recommended in the standard to obtain more accurate prediction.

## 5. Conclusions

Tests on corrosion fatigue propagation of the API 5L X56 pipeline in Bohai sea are carried out. To obtain the material constants in Paris law, the standard C(T) specimens made of API 5L X56 steel material in air and seawater are tested, respectively. The stress intensity factors in both C(T) specimens and in pipeline specimen are calculated from finite element analysis. Using Paris law, the fatigue life of submarine pipelines in Bohai sea is predicted. Based on the above studies, the following conclusions are drawn:

1. Based on finite element analysis to calculate the stress intensity factor of C(T) specimen, it is found that the recommended equation of shape factor in GB/T 6398-2000 produces relatively lower values. Especially for the shallow crack (a/W < 0.4), such shape factor provides prediction with a large error.
2. By comparing the Paris law constants obtained from equations in the standard and from the presented equation in this study, it is found that the Paris law constants calculated from the equation recommended in the standard are usually smaller than the results calculated from the new presented equation of the shape factor. When the fatigue life is predicted by using the equation recommended in the standard, the prediction is too conservative.
3. The datasets obtained from the FCG test show that for a given ΔK, the FCG rate (*da/dN*) in seawater is 1.6-times that in air and the effect of seawater on corrosion fatigue decreases with the increase of the FCG rate.
4. When using the finite element analysis to simulate the expansion of fatigue crack in the pipeline, the fatigue crack propagation starts to expand faster along the pipeline radial direction while the crack propagates relatively slowly along the circumferential direction in the early stage. When the crack depth increases to a certain extent, the expansion rate in the circumferential direction also increases. When the crack expands to a certain extent in the circumferential direction, the fatigue crack growth rate will decrease, which is in agreement with the experimental results.
5. Based on the combination of the FCG test and finite element simulation, Paris law can be used to predict fatigue crack growth of submarine pipeline accurately and effectively.

## Figures and Tables

**Table 1:** Impact energy for API 5L X56 steel material.

Test ID	Test Materials	Test Temperature (°C)	Impact Energy (J)
1	API 5L X56	20	158.27
2	API 5L X56	20	69.76
3	API 5L X56	20	124.66
4	API 5L X56	20	97.08
Mean Value	-	-	112.44

**Table 2:** Mechanical properties of API 5L X56 steel material.

Material	Elastic Modulus (GPa)	Yield Strength (MPa)	Poisson’s Ratio	$K_{IC}$ $\left(MPa\cdot \sqrt{m}\right)$
API 5L X56	200	340	0.3	188

**Table 3:** Parameters in fatigue crack propagation test.

Test ID	Environment	$a_{0}\;(\mathrm{mm})$	$a_{f}\;(\mathrm{mm})$	f (Hz)	*R*	$P_{\max}\;(\mathrm{kN})$
X56-A-1(CT)	Air	12.98	25.03	10	0.1	11
X56-S-1(CT)	Seawater	13.02	24.98	0.5	0.1	11
X56-S-2(CT)	Seawater	12.95	22.51	0.5	0.1	9.5
Submarine Pipeline Specimen	Seawater	5.5/52.1	13.9/60.2	0.5	0.1	235

**Table 4:** Relationship between crack length and back face strain.

Model ID	Crack Length/mm	Strain Value/με
X56-13	13	668
X56-14	14	763
X56-15	15	870
X56-16	16	992
X56-17	17	1131
X56-18	18	1290
X56-19	19	1472
X56-20	20	1684
X56-21	21	1930
X56-22	22	2218
X56-23	23	2558
X56-24	24	2965
X56-25	25	3456
X56-26	26	4055
X56-27	27	4803

**Table 5:** Stress-strain relation coefficient.

Equation	C_0_	C_1_	C_2_	C_3_	C_4_	C_5_	R^2^
Equation (8)	2.938	−2.060 × 10^−2^	−1.001 × 10^−5^	−2.935 × 10^−9^	−4.526 × 10^−13^	−2.802 × 10^−17^	0.99998
Equation (9)	0.973	−1685.761	−1.644 × 10^6^	8.214 × 10^9^	−7.381 × 10^12^	1	0.99999

**Table 6:** C(T) specimens material constants and remaining life.

Sample ID	Test Count	Calculation from Equation Recommended by GB/T 6398-2000					Calculation from Equation (11)				
		*C*	*m*	R^2^	Cycles	Error (%)	*C*	*m*	R^2^	Cycles	Error (%)
X56-A-1	29156	9.95 × 10^−9^	2.854	0.95	26283	−9.85	6.60 × 10^−9^	2.950	0.95	27851	−4.48
X56-S-1	15547	2.92 × 10^−9^	3.353	0.96	14159	−8.93	2.14 × 10^−9^	3.418	0.96	15273	−1.76
X56-S-2	16394	3.76 × 10^−8^	2.636	0.95	15358	−6.32	3.26 × 10^−8^	2.661	0.94	16154	−1.46
Pipeline	42079	3.76 × 10^−8^	2.636	0.95	38444/36651	-	3.26 × 10^−8^	2.661	0.94	40935/39383	-

**Table 7:** Radial crack propagation and loading cycles.

Model ID	Crack Depth	ΔK (mm/cycle)	$\mathrm{da}/\mathrm{dN}\;(MPa\cdot \sqrt{m})$	Cycles
Model-1-R	5.5	20.848	1.0538 × 10^−4^	-
Model-2-R	6	21.354	1.1220 × 10^−4^	4457
Model-3-R	7	23.006	1.3695 × 10^−4^	7302
Model-4-R	8	24.715	1.6636 × 10^−4^	6011
Model-5-R	9	25.776	1.8532 × 10^−4^	5396
Model-6-R	10	27.180	2.1341 × 10^−4^	4686
Model-7-R	11	27.714	2.2475 × 10^−4^	4449
Model-8-R	12	28.302	2.3767 × 10^−4^	4208
Model-9-R	13	29.561	2.6684 × 10^−4^	3748
Model-10-R	13.2	30.693	2.9490 × 10^−4^	678

**Table 8:** Circumferential crack growth and loading cycles.

Model ID	Crack Length	ΔK(mm/cycle)	$\mathrm{da}/\mathrm{dN}\;(MPa\cdot \sqrt{m})$	Cycles
Model-1-C	52.1	8.209	8.8265 × 10^−6^	-
Model-2-C	52.2	9.522	1.3099 × 10^−5^	3817
Model-3-C	52.5	11.539	2.1839 × 10^−5^	6868
Model-4-C	52.9	14.733	4.1841 × 10^−5^	4780
Model-5-C	53.5	17.073	6.1934 × 10^−5^	4844
Model-6-C	54.3	20.295	9.8105 × 10^−5^	4077
Model-7-C	55.3	22.699	1.3214 × 10^−4^	3784
Model-8-C	56.6	25.188	1.7429 × 10^−4^	3729
Model-9-C	58	27.199	2.1381 × 10^−4^	3274
Model-10-C	59.5	27.755	2.2564 × 10^−4^	4210
